# Key role of hydrogen in regulating hydrogenases and the reductive TCA cycle in a thermophilic, autotrophic sulfur-reducing bacterium

**DOI:** 10.1128/aem.01478-25

**Published:** 2025-11-11

**Authors:** Briana C. Kubik, James F. Holden

**Affiliations:** 1 Department of Microbiology, University of Massachusetts 196202, Amherst, Massachusetts, USA; Colorado School of Mines, Golden, Colorado, USA

**Keywords:** hydrogenase, reductive TCA cycle, thermophiles, autotrophs, hydrothermal vents, sulfur reduction

## Abstract

Many thermophilic autotrophs in deep-sea hydrothermal vents are anaerobes that require H_2_ for growth. However, our understanding of how non-methanogenic thermophilic hydrogenotrophs adapt to low H_2_ conditions is nascent. In this study, the thermophilic autotrophic thiosulfate-reducing bacterium *Desulfurobacterium thermolithotrophum* HR11 was grown at 72°C in a chemostat at 2 and 43 µM aqueous H_2_ concentrations to quantify changes in growth and H_2_S production kinetics and in its proteome in response to H_2_ availability. At 2 μM H_2_, cultures showed a decrease in specific growth rate, maximum cell concentration, and H_2_S production while cell yield (cells produced per mol of H_2_S produced) was unchanged. Differential proteomic analysis identified 79 and 66 proteins having more than twofold higher and lower abundances (*P*_adj_ < 0.01), respectively, under 2 μM H_2_ conditions out of 887 total detected proteins. Among these, a membrane-bound ferredoxin-dependent [NiFe] hydrogenase and a cytoplasmic NAD(P)^+^-dependent [NiFe] hydrogenase increased in cells grown on 2 μM H_2_. Also, on 2 μM H_2_, a membrane-bound menaquinone-dependent [NiFe] hydrogenase increased and likely generated a proton motive force and drove ATP synthesis, while a membrane-bound menaquinone-dependent [FeFe] hydrogenase and a membrane-bound NADH dehydrogenase decreased. Four reductive TCA cycle enzymes used for CO_2_ fixation were more abundant on 2 µM H_2_. These data suggest that hydrogenase and CO_2_ fixation enzyme abundances are regulated by H_2_ availability and support the idea of a shift in electron disposal toward CO_2_ reduction and assimilation on low H_2_.

**IMPORTANCE:**

Understanding how thermophilic, autotrophic sulfur-reducing bacteria such as *Desulfurobacterium thermolithotrophum* adapt to varying H_2_ concentrations is important for understanding competition and survival strategies in energy-limited subseafloor hydrothermal environments. In this study, *D. thermolithotrophum* showed metabolic adaptations under low H_2_ conditions that may provide them with a competitive growth advantage in hydrothermal vent ecosystems when H_2_ is limited and other thermophilic hydrogenotrophs, such as methanogens, are also commonly found. This work also demonstrated the dynamic relationship between different types of hydrogenases and how they are coordinated with each other and other key pathways such as CO_2_ fixation.

## INTRODUCTION

Thermophilic sulfur-reducing microbes play a central role in deep-sea hydrothermal vent ecosystems, where they contribute to sulfur and carbon cycling (1). Hydrothermal vents are characterized by steep chemical and thermal gradients and varying availability of key electron donors, such as H_2_ (2, 3). H_2_ availability in hydrothermal vents can vary significantly due to differences in host rock types and age of a hydrothermal system (2–5). *Desulfurobacterium* and *Thermovibrio* are common thermophilic autotrophic sulfur- and thiosulfate-reducing bacteria in hydrothermal vents (6–9) and are generally detected with other thermophilic hydrogenotrophs (e.g., methanogens) (10–15). This suggests that these hydrogenotrophs are competing for H_2_ in their natural habitats. In these environments, the ability of microorganisms to adapt to low H_2_ concentrations is important for their competition with other hydrogenotrophs and for their survival.

Research on H_2_-limited growth kinetics and physiological changes in hydrogenotrophs has primarily focused on methanogens, which show slower growth but higher cell yield (i.e., biomass produced per mole of CH_4_ produced) upon H_2_ limitation as a metabolic tradeoff (16–19). These methanogens also switched from using primarily a H_2_-dependent methylene-tetrahydromethanopterin (H_4_MPT) dehydrogenase to a coenzyme F_420_-dependent methylene-H_4_MPT dehydrogenase in their CO_2_ fixation pathway when grown on low H_2_ (17–19). Growth on low H_2_ coupled with decreasing Gibbs energy for the methanogenesis reaction also led to increased fractionation of carbon isotopes between CO_2_ and CH_4_ (19–21), which was proposed to be due to the low thermodynamic drive and reversal of the Wood-Ljungdahl methanogenesis pathway (20). Similarly, *Desulfurobacterium thermolithotrophum* HR11 showed a growth rate-growth yield metabolic tradeoff as growth rates decreased (22). No sulfide was detected when it was grown on low H_2_ relative to that on high H_2_ despite reaching similar cell concentrations. However, the effect of H_2_ limitation on the physiology of thermophilic autotrophic sulfur reducers is unknown.

In this study, *D. thermolithotrophum* HR11 was grown in a chemostat under high and low H_2_ conditions to quantify changes in specific growth rates, H_2_S production, and cell yields (cells produced per mole of H_2_S produced). The complete genome sequence of *D. thermolithotrophum* HR11 was determined by merging new long-read sequencing data with a previous draft genome sequence (23). The chemostat experiments were coupled with proteomic analyses to identify differentially abundant proteins. The goal was to determine if there is evidence of a physiological shift in *D. thermolithotrophum* HR11 that is indicative of a growth rate-growth yield metabolic tradeoff. This work contributes to a deeper understanding of the physiology of thermophilic thiosulfate reducers and their ecological roles and competitive dynamics in the subseafloor biosphere.

## RESULTS

### Genome sequencing

Whole genome sequencing of *D. thermolithotrophum* HR11 generated a total of 278,596 trimmed reads and 614,571,448 sequenced bases using Oxford Nanopore long-read sequencing. The genome assembly resulted in one complete circular genome sequence that was 1,562,359 bp long and one complete circular plasmid sequence that was 11,424 bp long. When Nanopore sequence data were combined with previous Illumina MiSeq data (23), the average genome coverage was >1,700-fold. The annotation resulted in 1,650 protein-coding genes in the chromosome.

### Growth kinetics

**Fig 1:**
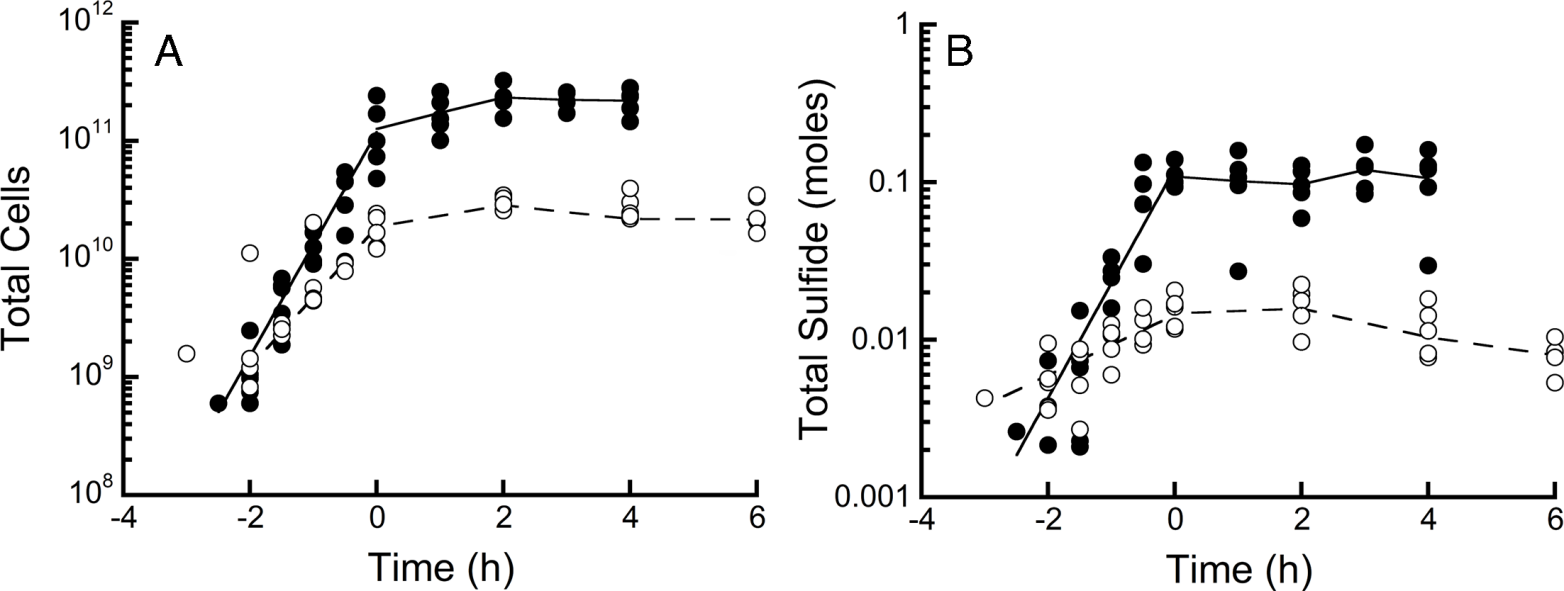
Growth analysis of *D. thermolithotrophum*. Total cell (**A**) and total sulfide (**B**) amounts per bioreactor for *D. thermolithotrophum* HR11 grown in batch phase and then in chemostat phase on 43 µM (●) and 2 µM (○) aqueous H_2_. The chemostat phase of growth started at time point 0.

The specific growth rate of *D. thermolithotrophum* HR11 in the batch phase of growth prior to starting the chemostat decreased significantly (*P* < 0.001) from 2.31 ± 0.29 h^−1^ (±95% confidence interval [CI], 18 min doubling time, *n* = 5) when grown on 43 µM H_2_ to 1.29 ± 0.13 h^−1^ (32 min doubling time, *n* = 5) when grown on 2 µM H_2_ (Fig. 1A). In the chemostat phase, the average total number of cells within the chemostat decreased significantly (*P* < 0.001) from 1.94 × 10^11^ cells when grown on 43 µM H_2_ to 2.51 × 10^10^ cells when grown on 2 µM H_2_ (Fig. 1A). The amount of H_2_S in the chemostat also decreased significantly (*P* < 0.001) from 107 ± 15 mmoles (±95% CI) on 43 µM H_2_ to 13 ± 2 mmoles on 2 µM H_2_ conditions (Fig. 1B). The average cell yields during chemostatic growth at 43 µM H_2_ and 2 µM H_2_ were 2.40 × 10^12^ cells/mol H_2_S and 2.21 × 10^12^ cells/mol H_2_S, respectively, and were not significantly different (*P* > 0.5) (Fig. S1).

### Differential proteomics

*D. thermolithotrophum* HR11 proteins were collected from four 43 μM H_2_ and four 2 μM H_2_ chemostats. A total of 887 unique proteins were detected using mass spectrometry, and 737 of these were tagged with isobaric markers for differential proteomic analysis. Principal component analysis (Fig. S2) and correlation analyses (Fig. S3) comparing each of the eight protein samples showed that the four 43 μM H_2_ protein samples (H1–H4) were highly similar and three of the four 2 μM H_2_ protein samples (L1–L3) were highly similar.

A negative binomial model was fit to the proteomic data to estimate changes in protein abundance and account for biological variability and replicate dispersion. The *P* values were adjusted for multiple testing to control the false discovery rate. Differential proteomics showed differential production of 145 proteins (average fold change > 2 and *P*_adj_ < 0.01, Fig. S4) with 79 having more than twofold higher abundance (Table S1) and 66 having more than twofold lower abundance (Table S2) on 2 µM H_2_ conditions compared to 43 µM H_2_ conditions. An additional 592 proteins were tagged but showed less than twofold differences in abundance or had a *P*_adj_ > 0.01 (Table S3), and 150 proteins were detected by mass spectrometry but were untagged (Table S4). Differences in protein abundances were examined with a particular focus on hydrogenases, reductive TCA cycle-associated proteins, NADH dehydrogenase, putative sulfur reduction-associated proteins, *c*-type cytochrome proteins, and membrane-bound ATP synthase.

**Fig 2:**
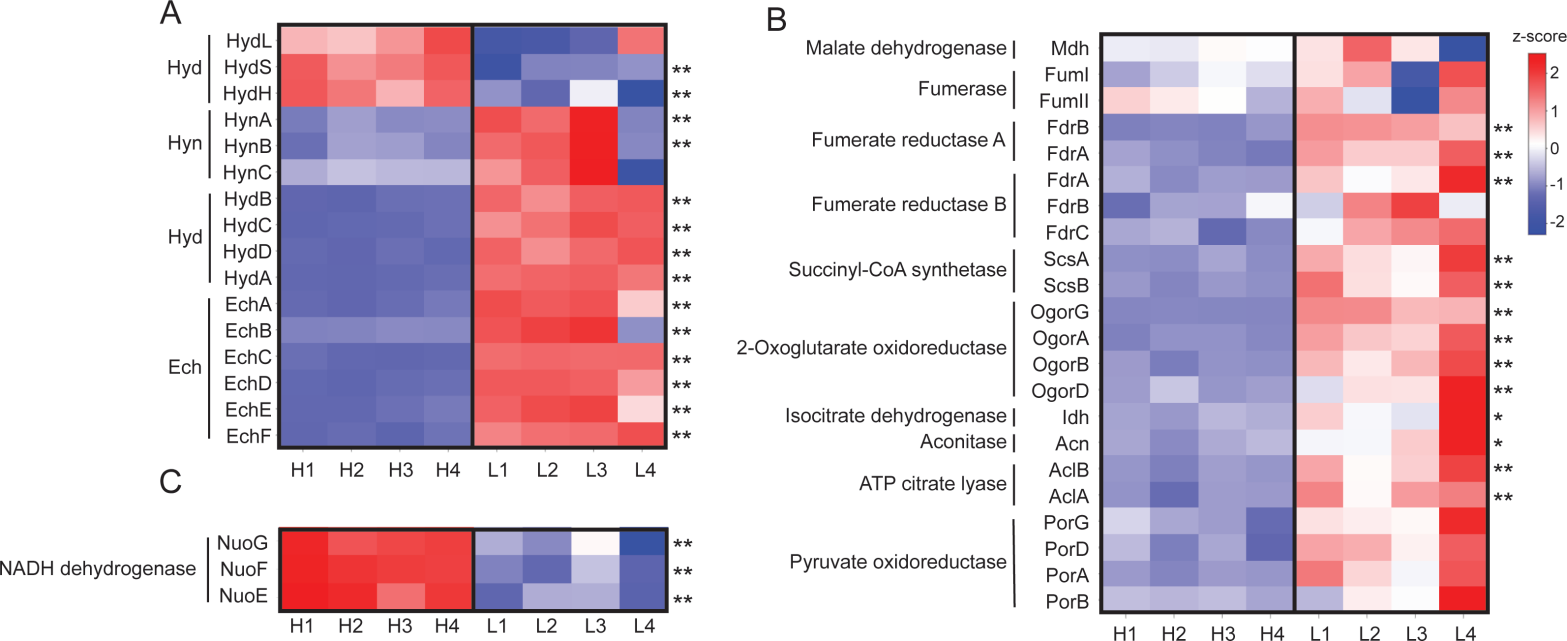
Proteomics heatmap of key proteins. Differential proteomic abundance analysis and heatmap of membrane-bound and cytoplasmic hydrogenase proteins (**A**), reductive TCA cycle-associated proteins (**B**), and NADH dehydrogenase proteins (**C**) in *D. thermolithotrophum* HR11 grown in high H_2_ (H1–H4) and low H_2_ (L1–L4) conditions. Relative protein abundances with more than twofold average differences between the two growth conditions and adjusted *P* values that are either <0.05 or <0.01 are shown with * and **, respectively.

**Fig 3:**
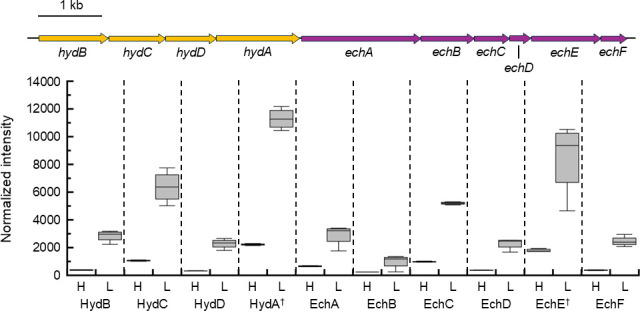
Proteomics results for NAD(P)^+^- and ferredoxin-dependent hydrogenases. Normalized proteome reporter ion intensities for the HydBCDA cytoplasmic NAD(P)H-dependent hydrogenase and the EchABCDEF membrane-bound ferredoxin-dependent hydrogenase for *D. thermolithotrophum* HR11 grown in high H_2_ (H) and low H_2_ (L) conditions. Proteins are identified by their subunit and shown as part of their encoding operons (drawn to scale). The box represents the interquartile range (IQR) extending from the first quartile (Q1) to the third quartile (Q3) with a solid line in the middle showing the median. Whiskers represent Q3 or Q1 ± 1.5 × IQR. † indicates the catalytic subunit of each hydrogenase.

The HR11 genome encodes an operon for a cytoplasmic NAD(P)^+^-dependent [NiFe] hydrogenase (*hydBCDA*, locus tag GFV12_RS04425-GFV12_RS04440) and an operon for a membrane-bound ferredoxin-dependent [NiFe] hydrogenase (*echABCDEF*, GFV12_RS04395-GFV12_RS04420) that are collocated 51 nucleotides apart on the same DNA strand. HydBCDA was estimated to be 10.5- to 17.7-fold more abundant in 2 µM H_2_ grown cells relative to 43 µM H_2_ grown cells (Fig. 2A and 3). Similarly, EchABCDEF were estimated to be 7.8- to 15.2-fold more abundant in 2 µM H_2_ grown cells relative to 43 µM H_2_ grown cells (Fig. 2A and 3). The HR11 genome also encodes two operons for membrane-bound menaquinone-dependent hydrogenases, specifically a [NiFe] hydrogenase (*hynABC*, GFV12_RS01850-GFV12_RS01860) and an [FeFe] hydrogenase (*hydLSH*, GFV12_RS00720-GFV12_RS00730). HynABC was estimated to be 2.5- to 3.1-fold more abundant on 2 µM H_2_ relative to 43 µM H_2_, but only HynAB had *P*_adj_ <0.01 (Fig. 2A). HydSH was estimated to be 2.2- to 2.7-fold less abundant on 2 µM H_2_, possibly indicating it has a low affinity for H_2_, while the catalytic subunit HydL remained unchanged (Fig. 2A). The genome also encodes an operon for a cytoplasmic coenzyme F_420_-like [NiFe] hydrogenase (*frhAGB*, GFV12_RS01870-GFV12_RS01880). However, FrhAGB was not detected in any of the proteomic samples.

Proteomic analyses showed that four rTCA cycle enzyme complexes were more abundant in 2 µM H_2_ grown cells compared to 43 µM H_2_ grown cells. The HR11 genome encodes for two fumarate reductase operons (*fdrABC*, GFV12_RS01890-GFV12_RS01900 and GFV12_RS01905-GFV12_RS01915). FdrAB for one of the two paralogs was estimated to be 8.1- and 18.0-fold more abundant in 2 µM H_2_ grown cells relative to 43 µM H_2_ grown cells (Fig. 2B). FdrA in the second fumarate reductase was estimated to be 2.4-fold more abundant on 2 µM H_2_ (Fig. 2B). The genome encodes for succinyl-CoA synthetase (*sucBA*, GFV12_RS07365-GFV12_RS07370) and 2-oxoglutarate oxidoreductase (*ogorDABG*, GFV12_RS07375-GFV12_RS07390) in operons that are collocated on the same DNA strand. SucAB and OgorABGD were all estimated to be 3.2- to 4.8-fold higher abundance in 2 µM H_2_ grown cells (Fig. 2B). The alpha and beta subunits of ATP citrate lyase (AclAB, GFV12_RS05570-GFV12_RS05575) were estimated to be 2.2- to 3.3-fold more abundant in 2 µM H_2_ grown cells (Fig. 2B). Isocitrate dehydrogenase (Idh, GFV12_RS05580) and aconitase (Acn, GFV12_RS05585) were estimated to be 3.0- and 2.5-fold more abundant on 2 µM H_2_ but were only significant at *P*_adj_ < 0.05 (Fig. 2B). There were no differences in the abundances of malate dehydrogenase (Mdh, GFV12_RS04390), either of two fumarases (FumI and II, GFV12_RS04385 and GFV12_RS04605), or pyruvate oxidoreductase (PorABGD, GFV12_RS02030-GFV12_RS02045) (Fig. 2B).

The HR11 genome encodes for a membrane-bound proton-translocating ATP synthase (*atpBE* and *atpFF′HAGDC*, GFV12_RS07710-GFV12_RS07715 and GFV12_RS06145-GFV12_RS06175). All the ATP synthase subunits were tagged and detected by mass spectrometry but did not change in relative abundance with H_2_ availability (Table S3). The genome also encodes for a membrane-bound NADH dehydrogenase complex in two separate operons (*nuoGFE* and *nuoABCDHIJKLMN*, GFV12_RS03465-GFV12_RS03475 and GFV12_RS05805-GFV12_RS05855). NuoGFE was estimated to be 3.2- to 3.7-fold less abundant in 2 µM H_2_ grown cells relative to 43 µM H_2_ grown cells (Fig. 2C). NuoC and NuoI were detected by mass spectrometry but were untagged (Table S4), while the remaining Nuo subunits were undetected.

For proteins related to sulfur and thiosulfate reduction, a putative NAD(P)H-dependent thioredoxin reductase (TrxR, GFV12_RS05945), disulfide reductase (TlpA, GFV12_RS05950), and NADH sulfur oxidoreductase (Nsr, GFV12_RS05415) did not show different abundances in 2 µM H_2_ grown cells compared to 43 µM H_2_ grown cells (Table S3). One of the nine putative *c*-type cytochrome proteins (GFV12_RS01210) was estimated to increase 2.2-fold in abundance and two other *c*-type cytochromes (GFV12_RS01165 and GFV12_RS02640) were estimated to decrease 3.4- and 4.8-fold in abundance on 2 µM H_2_ (Table S2). A putative peroxiredoxin (GFV12_RS03815), which protects cells from oxidative stress and acts as redox signaling regulators (24), was estimated to be 14.2-fold more abundant in low H_2_ grown cells relative to high H_2_ grown cells (Table S1).

The HR11 genome lacks genes for acetogenesis, namely phosphotransacetylase, acetate kinase, and ADP-forming acetyl-CoA synthetase. The genome encodes for an AMP-forming acetyl-CoA synthetase (GFV12_RS00945), which catalyzes the activation of acetate to acetyl-CoA (25). AMP-forming acetyl-CoA synthetase was estimated to increase in abundance 7.8-fold when cultures were grown on 2 µM H_2_ (Table S1).

## DISCUSSION

Understanding the growth and metabolic strategies of thermophilic hydrogenotrophs is necessary to predict the outcome of microbial competition in hydrothermal vent environments. In these habitats, where resources such as H_2_ can be scarce, the ability of hydrogenotrophic autotrophs to adapt their metabolism to low H_2_ flux conditions can also affect their survival. The growth rate-growth yield metabolic tradeoff represents an ecological strategy that optimizes microbial growth and competition as a function of nutrient availability. At high nutrient concentrations, microbes grow with high growth rates but low cell yields (lower biomass produced per substrate consumed), while under low nutrient conditions, they have lower growth rates but higher cell yields (higher biomass produced per substrate consumed) (26, 27). Hydrogenotrophic methanogens showed growth rate-growth yield metabolic tradeoffs with H_2_ availability by shifting their metabolism toward biomass production over energy generation in response to limited electron donor availability (16–19). A similar tradeoff was proposed for *D. thermolithotrophum* HR11 (22), and the proteomic shift with H_2_ availability toward CO_2_ fixation in this study supports this idea. A metabolic tradeoff in hydrogenotrophic sulfur reducers may negate any advantage gained by a metabolic tradeoff in hydrogenotrophic methanogens during competition between these organisms for a limited supply of H_2_.

**Fig 4:**
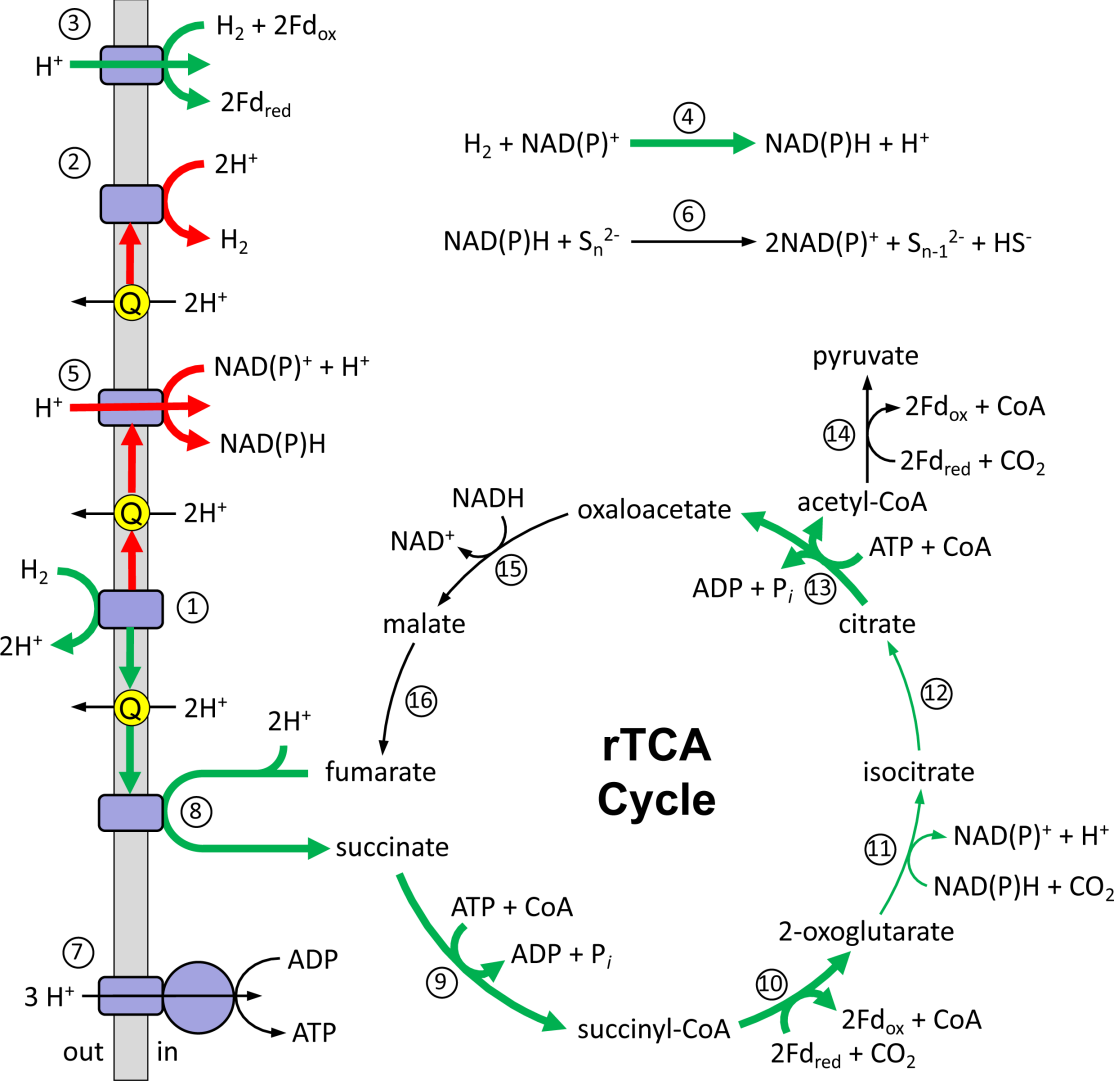
Model of *D. thermolithotrophum* metabolic tradeoff. Proposed metabolic pathway and metabolic tradeoff for *D. thermolithotrophum* HR11. The enzymes are (1) menaquinone-dependent [NiFe] hydrogenase (Hyn), (2) menaquinone-dependent [FeFe] hydrogenase (HydLSH), (3) ferredoxin-dependent [NiFe] hydrogenase (Ech), (4) NAD(P)^+^-dependent [NiFe] hydrogenase (HydBCDA), (5) NADH dehydrogenase, (6) NAD(P)H-dependent sulfur reductase, (7) ATP synthase, (8) fumarate reductase, (9) succinyl-CoA synthetase, (10) 2-oxoglutarate oxidoreductase, (11) isocitrate dehydrogenase, (12) aconitase, (13) ATP citrate lyase, (14) pyruvate oxidoreductase, (15) malate dehydrogenase, and (16) fumarase. CoA, coenzyme A; Fd, electron carrier ferredoxin; Q, menaquinone; rTCA cycle, reductive tricarboxylic acid cycle. Bold green and red arrows indicate a significant increase and decrease, respectively, in protein abundance on low H_2_ relative to high H_2_ conditions. Black arrows indicate no significant change in protein abundance on low H_2_ relative to high H_2_ conditions.

Hydrogenases catalyze the reversible conversion of H_2_ into protons and electrons and contribute to energy metabolism, redox balance, and electron transfer (Fig. 4). The HR11 genome encodes five different hydrogenase complexes, four of which were detected by proteomics in this study, demonstrating the organism’s diverse capacity for H_2_ utilization. Using the identification system of Greening et al. (28), *D. thermolithotrophum* HR11 has a group 1 membrane-bound HynABC [NiFe] hydrogenase and a group A membrane-bound HydLSH [FeFe] hydrogenase, both of which were detected by proteomics. Both hydrogenases likely use menaquinone-7 (MK-7H_6_) as an electron carrier, which is the predominant respiratory lipoquinone found in all other *Desulfurobacterium* species (8). HynABC, which increased in abundance on low H_2_, has a twin-arginine transport signal suggesting the catalytic subunit faces the periplasm leaving protons in the periplasm following H_2_ oxidation (Fig. 4). Menaquinone reduction and oxidation are coupled with proton translocation across the membrane, and both this and H_2_ oxidation by HynABC generate a proton motive force (Fig. 4). This proton gradient is likely used by membrane-bound ATP synthase, which was also detected by proteomics, for ATP generation via oxidative phosphorylation (Fig. 4).

*D. thermolithotrophum* HR11 also has a group 4 membrane-bound ferredoxin-dependent EchABCDEF hydrogenase and a group 3 cytoplasmic NAD(P)^+^-dependent HydABCD hydrogenase that were more abundant on 2 µM H_2_ than on 43 µM H_2_ in this study. They are collocated 51 nucleotides apart on the same DNA strand (Fig. 3) and may be co-regulated. These data suggest that *D. thermolithotrophum* shifted its use of H_2_ to directly producing reduced ferredoxin and NAD(P)H as sources of electrons for the cell when H_2_ was limited (Fig. 4).

The fate of the electrons carried by the menaquinones in the membrane appears to vary with H_2_ availability. In Kubik and Holden (22), *D. thermolithotrophum* HR11 grew slower and showed no H_2_S production under low H_2_ conditions in serum bottles while maintaining the same maximum cell concentrations relative to cells grown on high H_2_, which suggested a growth rate-growth yield metabolic tradeoff occurred in response to low H_2_ availability. Slow growth also occurred when thiosulfate was omitted from the growth medium and only CO_2_ was added as a terminal electron acceptor on both low and high H_2_ (22). In this study, HR11 altered its proteome in a manner suggesting a shift toward CO_2_ fixation when grown on limited H_2_.

The Gibbs energy of metabolic reactions is influenced by the concentration-dependent thermodynamic activity of electron donors and acceptors in the environment. H_2_ has a very negative midpoint potential (*E*_0_′ = −414 mV) making it an excellent electron donor (29). When H_2_ is abundant, *D. thermolithotrophum* HR11 likely oxidizes H_2_ using HynABC, passing electrons to menaquinones (*E*_0_′ = −74 mV) (29), then to membrane-bound menaquinol-oxidizing enzymes. The midpoint potential (*E*_0_′) of NAD(P)H is −320 mV (29), which means that electron transfer from menaquinol to form either NAD(P)H using NADH dehydrogenase or H_2_ using HydLSH is not thermodynamically favorable under standard equimolar conditions. High H_2_ conditions can drive electron transfer from menaquinol to NAD(P)H or H_2_, but under low H_2_ conditions, the thermodynamic drive for H_2_-dependent NAD(P)H or H_2_ production on the membrane via menaquinone intermediates is diminished.

NADH dehydrogenase in *D. thermolithotrophum* HR11 consists of 14 subunits with 11 subunits encoded in one operon (*nuoABCDHIJKLMN*) and three subunits in another operon (*nuoEFG*) (23). NuoAHJKLMN are embedded in the membrane and catalyze the oxidation of menaquinol while the remaining subunits in the complex (NuoBCDEFGI) face the cytoplasm and catalyze electron transfer with NAD(P)^+^ (30) (Fig. 4). In this study, NuoEFG showed a significant decrease in relative abundance on 2 µM H_2_ relative to 43 µM H_2_, while the remaining subunits were either detected but untagged or undetected in all samples. HydLSH also decreased in abundance on 2 µM H_2_. Therefore, *D. thermolithotrophum* HR11 appeared to shift the flow of electrons away from menaquinol-oxidizing NADH dehydrogenase and the [FeFe] hydrogenase under low H_2_ conditions (Fig. 4).

The proteomic results of this study also suggest that electrons from menaquinol shift toward CO_2_ fixation via the rTCA cycle under low H_2_ conditions (Fig. 4). The midpoint potential of fumarate/succinate is +30 mV (29), making fumarate a thermodynamically favorable electron acceptor for menaquinol oxidation. HR11 has two membrane-bound fumarate reductases that were higher in abundance when cultures were grown on 2 µM H_2_ relative to 43 µM H_2_. Furthermore, the rTCA cycle proteins succinyl-CoA synthetase, 2-oxoglutarate oxidoreductase, isocitrate dehydrogenase, aconitase, and ATP citrate lyase were more abundant on 2 µM H_2_ suggesting that CO_2_ fixation via the rTCA cycle and carbon assimilation was an alternate and favorable path for terminal electron accepting processes when *D. thermolithotrophum* was H_2_ limited (Fig. 4).

The thiosulfate reduction mechanism of *D. thermolithotrophum* HR11 is unknown. Its genome encodes homologs of NAD(P)H-dependent thioredoxin reductase (*trxR*) (31) and NAD(P)H-dependent sulfur reductase (*nsr*) (32) but lacks gene homologs for other mechanisms of sulfur metabolism such as a membrane-bound molybdopterin-dependent thiosulfate/polysulfide reductase (*sre*) (33), dissimilatory sulfite reductase (*dsr*) (34), sulfite oxidase (*sox*) (35), membrane-bound sulfide:quinone reductase (*sqr*) (32), and membrane-bound sulfane reductase (*mbs*) (36). Both TrxR and Nsr were detected by mass spectrometry in this study, but neither differed significantly in abundance on 2 µM versus 43 µM H_2_. Thiosulfate may be abiotically transformed to soluble polysulfide (S_*x*_^2−^) in the presence of sulfide at circumneutral pH (2 S_2_O_3_^2−^ + 4 HS^−^ + 8 H^+^ → S_8_^0^ + 6 H_2_O, then S_8_^0^ + HS^−^ → *n*S_*x*_^2−^ + H^+^) (37, 38). TrxR and Nsr, which are both cytoplasmic (31, 39), might then reduce the polysulfide to sulfide but require transport of the polysulfide into the cell. Furthermore, two putative membrane-bound *c*-type cytochrome proteins were more abundant on 2 µM H_2_ and may participate in the process of electron transfer to polysulfide.

The shift toward CO_2_ fixation on low H_2_ and the ability of *D. thermolithotrophum* HR11 to grow with only CO_2_ added as the terminal electron acceptor (22) raises questions about possible microbial metabolisms on the early Earth. During the early Archaean era (4.0–3.2 Ga), hydrothermal venting was abundant, but the global ocean was anoxic and ferruginous with limited availability of terminal electron acceptors (40). Isotopic evidence suggests sulfur reduction occurred ~3.5 Ga (41, 42), but intermediate sulfur species such as thiosulfate and S_8_^0^ would have been present in low micromolar concentrations during this time prior to photosynthetic ocean oxygenation and the Great Oxidation Event (40, 43, 44). Therefore, hydrothermally derived H_2_ and CO_2_ likely played a significant role in microbial metabolism, especially near hydrothermal vents. While hydrogenotrophic methanogenesis and acetogenesis were likely in this environment (40, 45), CO_2_ reduction to biomass alone should also be considered as a possible terminal electron-accepting process and energy generation mechanism on the early Earth. This entails H_2_ oxidation on the membrane by a hydrogenase coupled with menaquinone reduction, proton accumulation outside the cell via the outward-facing hydrogenase, and proton translocation via the menaquinone forming a proton motive force with electrons flowing into the rTCA cycle for CO_2_ reduction via fumarate reductase (Fig. 4). Sulfur reduction could provide additional energy for a cell by providing an additional electron sink if H_2_ and reducible sulfur were present.

In conclusion, these findings contribute to our understanding of microbial metabolism, especially when H_2_ is limited, in a thermophilic thiosulfate-reducing bacterium that is commonly found in deep-sea hydrothermal vents. The ability of *D. thermolithotrophum* HR11 to adapt to H_2_ limitation highlights its strategies for survival and competition with other hydrogenotrophs. This study demonstrated the coordination of its hydrogenases in response to environmental changes and the role of H_2_ in modulating the metabolism of *D. thermolithotrophum*. If and how H_2_ regulates gene expression in *D. thermolithotrophum* HR11 and the mechanism for thiosulfate reduction are the subjects of future studies.

## MATERIALS AND METHODS

### Growth medium and microorganism used

*D. thermolithotrophum* HR11 (DSM 100454) (14, 23) was obtained from Deutsche Sammlung von Mikroorganismen und Zellkulturen. All chemical reagents were purchased from Sigma-Aldrich. The growth medium was based on DSM medium 282 (22, 46) and is composed of the following per liter: 30 g of NaCl, 4.1 g of MgCl_2_∙6H_2_O, 3.40 g of MgSO_4_∙7H_2_O, 0.33 g of KCl, 0.25 g of NH_4_Cl, 0.14 g of CaCl_2_∙2H_2_O, 0.14 g of K_2_HPO_4_, 10 mL of DSM medium 141 trace elements solution, 10 mL of 141 vitamins solution, 0.1 mL of 0.1% (wt/vol) Na_2_SeO_4_ solution, 1 g of NaHCO_3_, 1 g of Na_2_S_2_O_3_, and 50 µL of 0.5% (wt/vol) resazurin. The medium was pH balanced to 6.00 ± 0.05 and reduced with 0.025% (wt/vol) cysteine-HCl.

*D. thermolithotrophum* HR11 was grown in a chemostat as previously described (14) to measure growth and H_2_S production kinetics and generate biomass for proteomic analysis. When previously grown in a chemostat, the Monod kinetic H_2_ half-saturation constant (*K*_s_) and minimum H_2_ concentration for the growth *D. thermolithotrophum* HR11 were 30 and 3 µM, respectively (14). In this study, 1.8 L of growth medium was added to a 2 L bioreactor (Ace Glass) and stirred at ~180 rpm. The growth medium was heated to 72 ± 0.1°C and the pH was maintained at pH 6.0 ± 0.1 by the automatic addition of 0.5 mM HCl. Gas flow rates were established prior to the experiment using a bubble meter and blended using a mass flow controller (Matheson Tri-Gas). Mixed gases were added to the bioreactor through a single submerged fritted bubbler (porosity B [70–100 μm]; ASTM certified; Ace Glass). For high H_2_ conditions, the H_2_ flow rate was set at 120 mL/min and the CO_2_ flow rate at 30 mL/min. For low H_2_ conditions, the H_2_:N_2_ (5%:95%) flow rate was set at 60 mL/min, the N_2_ flow rate at 60 mL/min, and the CO_2_ flow rate at 30 mL/min. The aqueous H_2_ concentration in the bioreactor prior to inoculation averaged 43 µM for the high H_2_ condition and 2 µM for the low H_2_ condition. Eight liters of sterile growth medium was placed in a 12 L glass carboy and pH balanced to 6.00 ± 0.05 using 5 M HCl. The reservoir growth medium was heated to 72°C, degassed with N_2_ at a flow rate of 30 mL/min, and reduced with 0.025% cysteine-HCl.

The bioreactor was inoculated with 10 mL of *D. thermolithotrophum* HR11 that was in logarithmic growth phase. Time points were taken throughout the growth of the organism and preserved in 25 µL of 37% (vol/vol) formaldehyde for cell counts using a Petroff-Hausser counting chamber and phase-contrast light microscopy. The H_2_ and H_2_S concentrations in the bioreactor headspace were measured throughout growth using a gas chromatograph equipped with a thermal conductivity detector (TCD) and a HayeSep D packed column (Supelco, 6′ × ⅛″ stainless steel) for H_2_ detection and a flame photometric detector (FPD) and a DB-1 type MXT-1 capillary column (Restek, 60 m × 0.53 mm I.D. 5.0 µm) for H_2_S detection. The aqueous H_2_ and H_2_S concentrations were measured by draining 25 mL of the bioreactor fluid at various time points directly into anoxic 60 mL serum bottles that were flushed with N_2_ and measuring the headspace H_2_ using gas chromatography and the dissolved H_2_S (after addition of 10 M NaOH) using the methylene blue method (47). When the cultures reached their maximum cell concentration, the bioreactor was switched to chemostat mode where the influent and effluent flow were equal and set to the growth rate of the culture using a dual-channel peristaltic pump (Ismatec). Growth of HR11 stabilized soon after the chemostat was started and was monitored for at least three volume replacements to collect kinetic data. Five replicates were taken for each of the two conditions.

Growth parameters, including specific growth rate, maximum cell concentration, cell-specific H_2_S production rate, and H_2_S production-based cell yield, were calculated for each condition. The growth rate of HR11 was determined by fitting an exponential curve to the cell concentration data contained in the logarithmic portion of growth while in batch phase. The H_2_S production-based cell yield was calculated by dividing the total number of cells in the bioreactor by the total moles of H_2_S in the bioreactor at the final chemostat time point.

### Genome sequencing

High molecular weight DNA from *D. thermolithotrophum* HR11 was extracted using a DNeasy Blood & Tissue kit (Qiagen). Following extraction, Oxford Nanopore long-read sequencing was used for whole genome sequencing. Sample libraries were prepared using the PCR-free Ligation Sequencing kit (Oxford Nanopore Technologies) with the NEBNext Companion Module (New England Biolabs) per the manufacturer’s protocol. Both Nanopore library construction and sequencing were performed by SeqCenter (Pittsburgh, PA, USA). Nanopore sequencing was performed on an Oxford Nanopore GridION sequencer using R10.4.1 flow cells in one or more multiplexed shared-flow-cell runs. Run design utilized the 400 bps sequencing mode with a minimum read length of 200 bp. Adaptive sampling was not enabled. Guppy version 6.5.7 (https://pypi.org/project/ont-pyguppy-client-lib/6.5.7) was used for super-accurate basecalling, demultiplexing, and adapter removal. No quality trimming was performed.

*De novo* genome assemblies were generated from the Nanopore read data with Flye (48) under the nano-hq (ONT high-quality reads) model. Additional Flye options initiated the assembly by first using reads longer than an estimated *N*_50_ based on a genome size of 6 Mbp. Subsequent polishing used the Illumina read data from Holden et al. (23) with Pilon (49) under default parameters. To reduce erroneous assembly artifacts caused by low-quality Nanopore reads, long read contigs with an average short read coverage of 15 or less were removed from the assembly. Assembled contigs were evaluated for circularization via Circlator (50) using the Nanopore long reads. The completeness of the genome sequence was determined using CheckM (51). Open reading frames were identified and annotated using the NCBI Prokaryotic Genome Annotation Pipeline version 6.7.

### Proteomic analyses

Total protein was extracted from 8 cell pellets, 4 from 43 µM H_2_ conditions and 4 from 2 µM H_2_ conditions. At the end of the chemostat run, the remaining 1.5 L of liquid in the bioreactor was concentrated by centrifugation at 10,000 × *g* at 4°C for 60 min and washed two times with 50 mM Tris-HCl (pH 8.0). The harvested cell pellet was stored at −20°C in a 1.5 mL Eppendorf tube until further use. Cell lysis was performed by thawing and sonicating the cell pellets. Cell lysis was confirmed using phase-contrast light microscopy. Protein extraction, reduction, alkylation, trypsin digestion, and cleanup were performed using the EasyPep Mini MS Sample Prep Kit (Thermo Scientific). Protein concentrations were determined using the DC protein assay kit (BioRad) with bovine serum albumin as a protein standard.

Eight isobaric tags from the TMT 10plex isobaric label reagent kit (Thermo Scientific) were used to label digested peptides from each of the eight samples. The labeled peptide samples were combined in equal amounts (15 µg), resulting in one sample containing eight isobarically labeled samples (four high H_2_ and four low H_2_). The combined samples were analyzed two times (instrumental technical replicates) using a Thermo Easy-nLC 1000 nanoscale liquid chromatography (nanoLC) system at the Mass Spectrometry Center at the University of Massachusetts Amherst, as described in Kashyap and Holden (52). Briefly, 5 µL of sample was loaded onto a trap column and desalted with 15 µL buffer A (0.1% [vol/vol] formic acid in water). Peptides were then eluted through a 50 cm nanocolumn (PepMap RSLC, 500 mm × 75 µm, Thermo) over 180 min using a gradient from 5% buffer B (0.1% formic acid in acetonitrile) to 45% buffer B at a flow rate of 300 nL/min into the mass spectrometer. Eluted peptides were detected with an Orbitrap Fusion (Thermo Electron). MS1 spectra were acquired at 120,000 resolution over a range of 375–1,500 *m/z* with a 2 s cycle time. Data-dependent tandem mass spectrometry (MS/MS) spectra for peptide identification were acquired in the linear ion trap using an isolation width of 1.2 *m/z* and collision-induced dissociation with normalized collision energy (NCE) of 35%. TMT quantification was achieved by synchronous precursor selection (SPS) of the top 5 precursors (MS1 charge state, +2; isolation width, 1.3 *m/z*) or top 10 precursors (MS1 charge state, +3; isolation width, 0.7 *m/z* or MS1 charge state, +4 to +6; isolation width, 0.5 *m/z*) and subsequent MS^3^ using high-energy collisional dissociation at 65% NCE with Orbitrap detection at 50,000 resolution.

The Proteome Discoverer v2.3 software was used to process the RAW instrument files from mass spectrometry. Proteins were identified using the SEQUEST HT analysis program and the *D. thermolithotrophum* HR11 proteome (GenBank accession number CP176813). The search was configured with static modifications for the TMT reagents (+229.163 Da) on lysines and N termini, carbamidomethyl (+57.021 Da) on cysteines, dynamic modifications for oxidation of methionine residues (+15.995 Da), precursor mass tolerance of 10 ppm, fragment mass tolerance of 0.6 Da, and trypsin cleavage with a maximum of two missed cleavage sites. A reversed sequence decoy strategy was used to control peptide false discovery, and identifications were validated by Percolator software. Only peptides with *q* values of ≤0.05 were used, and at least one unique peptide was required for reporting identified proteins. Reporter ions were corrected for isotopic impurities (specific to the lot of label reagents provided by Thermo Scientific) and quantified with a coisolation threshold of 75, and SPS mass matches a threshold of 65%. Protein abundances were determined by summing up the reporter ion intensities by channel and values were normalized to the most abundant channel.

Normalized protein abundances were used to identify differentially produced proteins using the DEBrowser package in R (53). Proteins for which the maximum intensity across all conditions was less than 10 were filtered out. The data were normalized using the Trimmed Mean of *M* values in the edgeR package (54). edgeR estimates biological variability using an empirical Bayes approach, fits a negative binomial generalized log-linear model to the data, and performs protein-wise statistical tests to assess differential abundance between conditions. The *P* values were adjusted for multiple testing using the Benjamini-Hochberg method to control the false discovery rate. Proteins were considered differentially produced if |log_2_FoldChange| > 1 and the adjusted *P* values were <0.01.

## Data Availability

The whole-genome sequence project was deposited at DDBJ/ENA/GenBank under accession numbers CP176813 and CP176814. The raw reads were deposited in the Sequence Read Archive under BioProject number PRJNA580254. The mass spectrometry proteomics data were deposited with the ProteomeXchange Consortium (http://proteomecentral.proteomexchange.org) via the PRIDE partner repository (55) and assigned the project accession number PXD070557. Data used for differential protein analyses are included in Tables S1 to S4 in the supplemental material.

## SUPPLEMENTAL MATERIAL

Supplemental figures aem.01478-25-s0001.pdf Fig. S1 to S4.

Supplemental tables aem.01478-25-s0002.xlsx Tables S1 to S4.

ASM does not own the copyrights to Supplemental Material that may be linked to, or accessed through, an article. The authors have granted ASM a non-exclusive, world-wide license to publish the Supplemental Material files. Please contact the corresponding author directly for reuse.
