# Supplementary material for: Key role of hydrogen in regulating hydrogenases and the reductive TCA cycle in a thermophilic, autotrophic sulfur-reducing bacterium
Source: Appl Environ Microbiol. 2025 Nov 11;91(12):e01478-25. doi: 10.1128/aem.01478-25 (PMC12724263; doi:10.1128/aem.01478-25)
Supplement: Supplemental figures — Fig. S1 to S4. [file aem.01478-25-s0001.pdf]

Supplemental Material for

Key role of hydrogen in regulating hydrogenases and the reductive TCA cycle in a thermophilic, autotrophic sulfur-reducing bacterium

Briana C. Kubik[1], James F. Holden[1]*

[1]Department of Microbiology, University of Massachusetts, Amherst, MA, USA.

*Address correspondence to James F. Holden, jholden@umass.edu

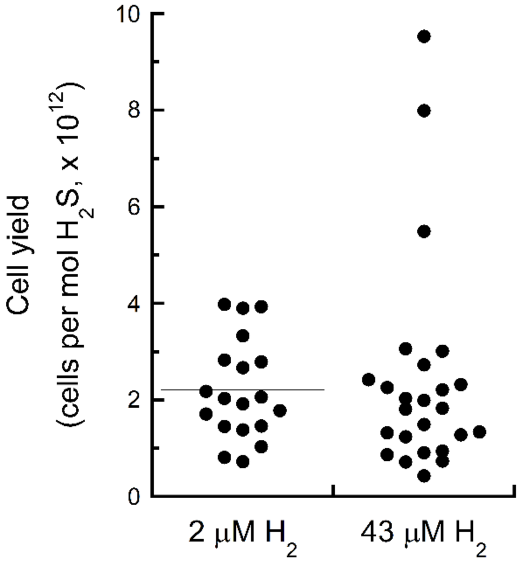

Fig. S1 | Cell yields for *D. thermolithotrophum* HR11 when grown in a chemostat at 2 μM H_2 and 43 μM H_2.

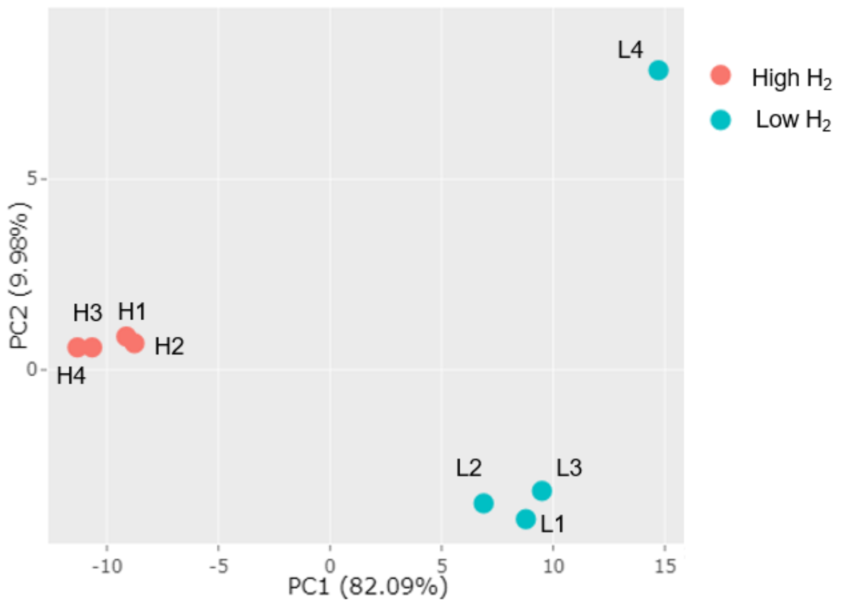

Fig. S2 | Principal component analysis (PCA) for proteomic samples grown in high H_2 (H1-H4) and low H_2 (L1-L4) conditions.

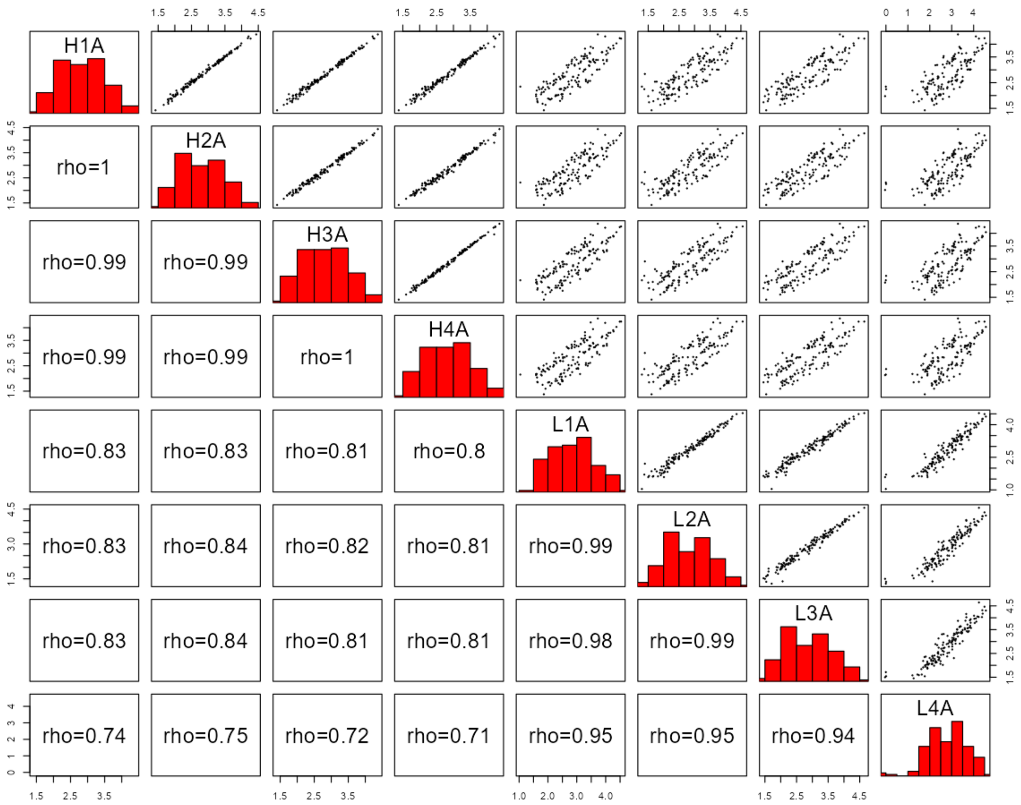

Fig. S3 | Correlation plots for proteomic samples grown in high H_2 (H1-H4) and low H_2 (L1-L4) conditions.

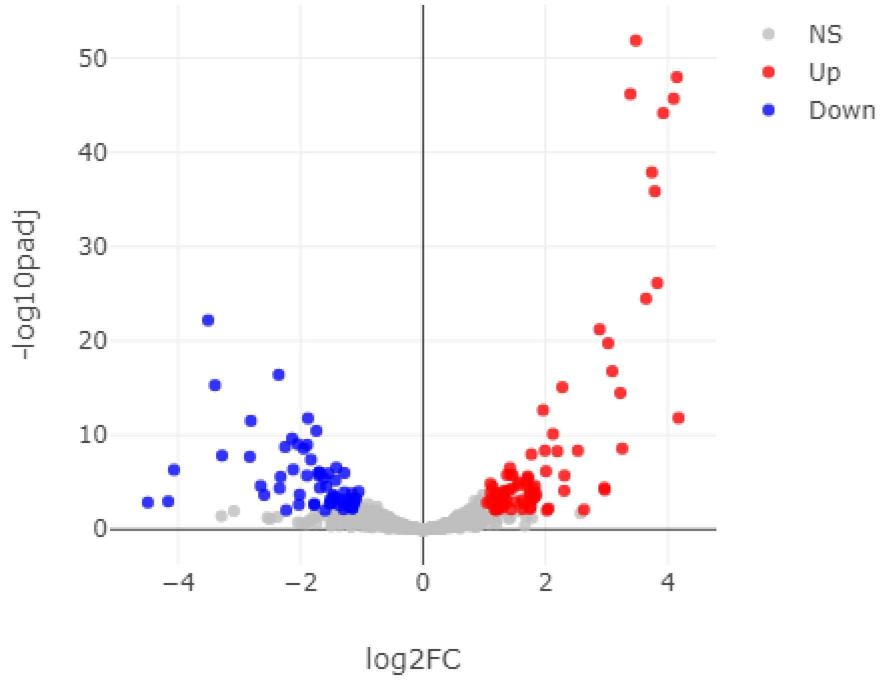

Fig. S4 | Volcano plot of differentially abundant proteins in *D. thermolithotrophum* HR11. A volcano plot showing 79 proteins with higher abundances (●) and 66 proteins with lower abundances (●) on low H_2 conditions relative to high H_2 conditions. To be differentially abundant, proteins had to be at least two-fold more abundant in one condition relative to the other condition with an adjusted *p*-value < 0.01. Protein abundance ratios in grey (●) were not significantly different.
